# Unravelling the hidden heterogeneities of diffuse large B-cell lymphoma based on coupled two-way clustering

**DOI:** 10.1186/1471-2164-8-332

**Published:** 2007-09-22

**Authors:** Wei Zhang, Li Li, Xia Li, Wei Jiang, Jianmin Huo, Yadong Wang, Meihua Lin, Shaoqi Rao

**Affiliations:** 1 The First Clinical College, Department of Bioinformatics, and the Bio-pharmaceutical Key Laboratory of Heilongjiang Province and State, Harbin Medical University, Harbin 150086, China; 2 Institute of Medical Genetics, Tongji University, Shanghai 200092, China; 3 Department of Computer Science, Harbin Institute of Technology, Harbin 150080, China; 4 The Biomedical Engineering Institute, Capital Medical University, Beijing 100054, China; 5 Department of Molecular Cardiology, Cleveland Clinic, Cleveland, OH 44195, USA

## Abstract

**Background:**

It becomes increasingly clear that our current taxonomy of clinical phenotypes is mixed with molecular heterogeneity. Of vital importance for refined clinical practice and improved intervention strategies is to define the hidden molecular distinct diseases using modern large-scale genomic approaches. Microarray omics technology has provided a powerful way to dissect hidden genetic heterogeneity of complex diseases. The aim of this study was thus to develop a bioinformatics approach to seek the transcriptional features leading to the hidden subtyping of a complex clinical phenotype. The basic strategy of the proposed method was to iteratively partition in two ways sample and feature space with super-paramagnetic clustering technique and to seek for hard and robust gene clusters that lead to a natural partition of disease samples and that have the highest functionally conceptual consensus evaluated with Gene Ontology.

**Results:**

We applied the proposed method to two publicly available microarray datasets of diffuse large B-cell lymphoma (DLBCL), a notoriously heterogeneous phenotype. A feature subset of 30 genes (38 probes) derived from analysis of the first dataset consisting of 4026 genes and 42 DLBCL samples identified three categories of patients with very different five-year overall survival rates (70.59%, 44.44% and 14.29% respectively; *p *= 0.0017). Analysis of the second dataset consisting of 7129 genes and 58 DLBCL samples revealed a feature subset of 13 genes (16 probes) that not only replicated the findings of the important DLBCL genes (e.g. *JAW1 *and *BCL7A*), but also identified three clinically similar subtypes (with 5-year overall survival rates of 63.13%, 34.92% and 15.38% respectively; *p *= 0.0009) to those identified in the first dataset. Finally, we built a multivariate Cox proportional-hazards prediction model for each feature subset and defined *JAW1 *as one of the most significant predictor (*p *= 0.005 and 0.014; hazard ratios = 0.02 and 0.03, respectively for two datasets) for both DLBCL cohorts under study.

**Conclusion:**

Our results showed that the proposed algorithm is a promising computational strategy for peeling off the hidden genetic heterogeneity based on transcriptionally profiling disease samples, which may lead to an improved diagnosis and treatment of cancers.

## Background

When a patient is diagnosed with cancer, various clinical parameters are used to assess the patient's risk profile. However, the patients with a similar prognosis frequently respond very differently to the same treatment. This may occur because two apparently similar tumours are actually completely different diseases at the molecular level, often called genetic heterogeneity. It describes the biological complexity whereby apparently similar inheritable characters result from different genes or different genetic mechanisms. The presence of such heterogeneity has a significant impact on both the efficiency of modern clinical practice and biomedical research of common human diseases. Gene chip technology measuring the transcriptional omics holds a promise in tackling the heterogeneity issues for complex human diseases, i.e., the subtypes of a disease can be discovered accurately at a molecular level by analysis of the gene expression profiles. Recent examples can be witnessed in the studies of leukaemia [1,2], breast cancer [3,4], renal allograft [5], lung cancer [6,7] and prostate cancer [8], based on unsupervised hierarchical clustering. Diffuse large B-cell lymphoma (DLBCL) analyzed in this study is the most common type of lymphoma in adults and demonstrates very apparently clinical heterogeneity. It can be treated by chemotherapy in only approximately 40% of patients. Several recent studies used DNA microarrays to study DLBCL, suggesting that it is possible to identify subgroups of patients in terms of different survival courses via gene expression data [9,10], which are unlikely to be discovered by traditional clinical approaches.

However, most of the methods for peeling off heterogeneities resort to the unsupervised learning techniques, such as hierarchical clustering, to identify clinically relevant subtypes based on all genes or a large number of genes on microarrays. Their utility is limited when the disease heterogeneity is resulted from only a small subset of the genes that participate in a particular cellular process, leading to different clinical outcomes. When the full dataset is analyzed, the "signal" of this process may be completely overwhelmed by the "noise" generated by the vast majority of unrelated data. In this study, we thus proposed an improved heterogeneity analysis strategy over the coupled two-way clustering algorithms [11-13]. In the proposed two-way clustering algorithm, super-paramagnetic clustering (SPC) algorithm [13,14] was used to take its advantages as an efficient partitioner: the number of clusters was achieved by the algorithm internally, without a need to be externally prescribed; and its stability against noise, thus providing a mechanism to identify robust stable phenotypic clusters using the most compacted subset(s) of gene signatures that leads to the best fits of the sample partitions. The rapidly accumulated multiple lines of evidence from, among others, gene expression and protein-protein interaction studies, support that genes express and perform their highly integrated cellular functions in modular fashions in cells [15-17]. Also inspired by our recent success in peeling off the hidden genetic heterogeneities of cancers based on disease relevant functional modules [18], we further defined a GeneOntology (GO)-based [19-21] conceptual functional similarity measure in order to establish a functional validation for the identified gene subsets. Finally we proved the differential survival outcomes of new subtypes using Kaplan-Meier survival analysis and multivariate Cox proportional-hazards prediction modelling according to their clinical data. We demonstrated the behaviours and properties of the proposed method by applying it to two publicly available microarray datasets of diffuse large B-cell lymphoma (DLBCL), a notoriously heterogeneous phenotype.

## Results

### Description of DLBCL datasets

In this study, we used two published gene expression data for DLBCL. The first dataset, analyzed initially by Alizadeh et al. [9], consists of 42 samples, and 40 of them have survival data as well. The microarray data, available at Lymphoma/Leukemia Molecular Profiling Project [22], the website companion to [9], have expression profiles for 4026 genes, and among the 4026 genes, 1980 genes have missing values. We imputed missing values by the *K*-Nearest Neighbours method (*K *= 5) [23]. The second dataset, analyzed initially by Shipp et al [24] and available at the website for The Broad Institute's Cancer Program Data Sets [25], consists of 58 samples and 7129 genes.

### Coupled two-way clustering

We searched significant gene subsets using the well established coupled two-way clustering (CTWC) algorithm as implemented in a public sever [26], which used SPC as the underlying clustering tool to break down the total dataset into subsets of genes and samples iteratively until significant partitions (submatrices) were revealed. First, we clustered all samples using all genes to identify stable sample partitions and clustered all genes using all samples to identify stable gene subsets. Then, we clustered the genes gained in the previous step using the newly defined sample partitions (including all samples) to find the responsible gene subsets of high discriminating power. Finally, we clustered each sample partition again using each gene subset with high discriminating power. In the searching process, we explored the cluster depth for both dimensions of samples and genes. The cluster depth selected was based on the empirical judgement whether the clinical samples could be well separated using the candidate gene subset(s). For the dataset of Alizadeh et al [9], we stopped the sample clustering at the cluster depth of one, with eight stable and significant gene subsets (*G*_2_, *G*_3_, ..., *G*_9_) identified. For the dataset of Shipp et al [24], we also stopped the sample clustering at the cluster depth of one, with 30 stable and significant gene subsets (*G*_2_, *G*_3_, ..., *G*_31_) identified.

### Computing the functional concept consistency scores for the identified gene subsets

Co-expression genes often share some functional relevance. Because we clustered the samples based on expression similarity (and difference) among the putative feature genes, the samples within a cluster were expected to be more similar in transcriptional activities than those in different clusters. Hence, the sample partitions might reflect their differences in response to the underlying biology pathway(s) leading to the phenotypic differentiation. In order to establish the functional validation for the identified gene subsets, we defined a GeneOntology (GO)-based [19-21] conceptual functional similarity measure, called concept consistency score (see Methods for detail).

The CTWC algorithm was able to identify numerous highly correlated gene subsets during the recursive partitioning of samples and genes. In this study, our goal was to find some partition of DLBCL with high medical implications. The consistency scores for eight stable gene subsets (*G*_2_, *G*_3_, ..., *G*_9_) identified in the dataset of Alizadeh et al [9] were 1.00, 0.00, 0.11, 0.46, 0.30, 0.00, ∅ and 0.00, respectively. An empty value ∅ occurred because some genes had neither functional annotation nor a common parent node in GO. Subset *G*_2 _had the highest consensus score, and was thus selected as a modular signature for subtyping the disease samples. The consistency score of *G*_4 _was the highest (score = 0.48) among 30 stable gene subsets (*G*_2_, *G*_3_,, *G*_31_) identified in the dataset of Shipp et al [24]. Their scores were 0.44, 0.25, 0.48, 0.00, 0.41, 0.00, 0.39, 0.10, ∅, 0.38, 0.37, 0.00, 0.33, 0.32, 0.11, 0.05, 0.28, 0.27, 0.42, 0.24, 0.20, 0.19, 0.11, 0.01, 0.16, 0.15, 0.00, 0.11, 0.1, 0.31, respectively.

### Clustering samples by SPC, using the gene subsets with highest score as the modular features

**Figure 1:**
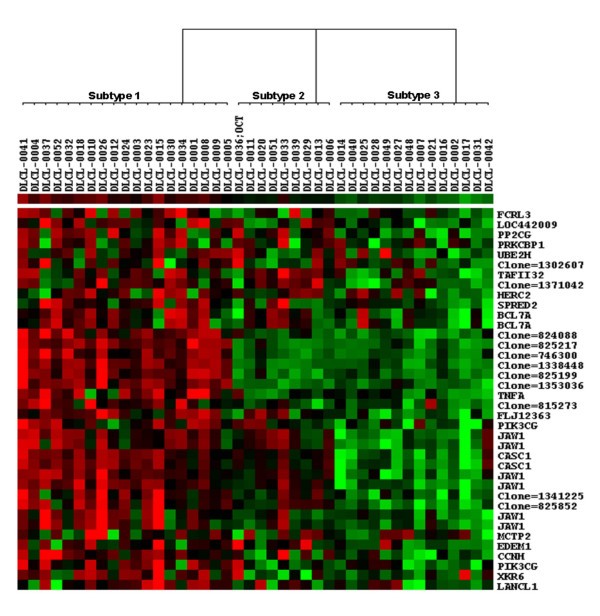
**The three partitions of DLBCL were identified using *G*_2 _as the disease feature set in the Alizadeh et al's dataset**. In the figure, each gene corresponds to a row, and each DLBCL sample corresponds to column. Forty-two DLBCL samples were divided into three subtypes (Subtype 1, Subtype 2 and Subtype 3). Red areas indicate increased expression, and green areas decreased expression. Genes that are characteristically expressed in three subtypes of diffuse large-B-cell lymphomas are indicated. The dendrogram at the top shows the degree to which each DLBCL subtype is related to the others with respect to gene expression.

When clustering a dataset using a subset of genes, it is important to know if the samples can be well characterized using such a subset. For the dataset of Alizadeh et al, forty-two DLBCL samples were clustered using the genes included in *G*_2 _using SPC, with the Euclidean distance and Pearson's correlation coefficient as the sample and the gene expression similarity measures, respectively. Figure 1, plotted by Treeview [27,28], shows clearly a partition of three subtypes of diffuse large-B-cell lymphomas for 42 patients per the expression patterns of 38 probes (representing a total of 30 unique genes/loci). Among 38 probes, 27 were annotated in GO: 6 were for the major lymphoid-restricted membrane protein (*JAW1*), 2 for the B-cell CLL/lymphoma 7A (*BCL7A*), 2 for phosphoinositide-3-kinase, catalytic, gamma polypeptide (*PIK3CG*) and 2 for cancer susceptibility candidate 1 (*CASC1*). In addition, *BCL7A *and *TNFA *were previously reported as the prognostic factors for lymphoma. Five of the remaining 11 probes were known transcribed loci. A careful scrutiny of *G*_2 _revealed that it largely captured complex modular activities that regulate cell cycling, DNA synthesis and repair, leukocyte adhesion, cell-cell signalling etc. and that mainly take place in nucleus and intracellularly. It is also interesting to note that *G*_2 _contained an "integral to plasma membrane" component consisting of the well known DLBCL relevant pathway – G-protein coupled receptor protein signalling pathway (e.g. *LANCL1*, *CASC1 *and *PIK3CG*) [29] and *JAW1 *for vesicle targeting and homocyte development. For the functional annotations for the known genes included in *G*_2_, see Additional File 1.

**Figure 2:**
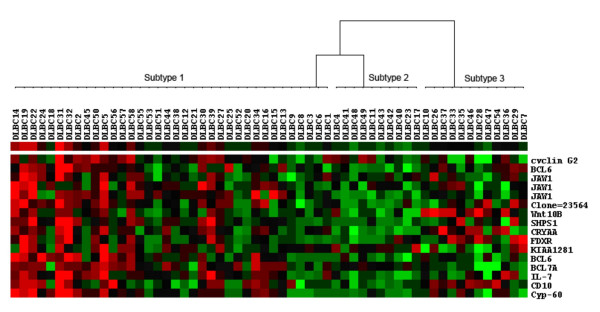
**The three partitions of DLBCL were identified using *G*_4 _as the disease feature set in the Shipp et al's dataset**. In the figure, 58 DLBCL samples were divided into three subtypes (Subtype 1, Subtype 2 and Subtype 3).

For the second DLBCL dataset, 58 DLBCL samples were clustered using the genes included in *G*_4 _using SPC. We again identified a partition of three subtypes of DLBCL among 58 patients per the expression patterns of 16 probes, as shown in Figure 2. Among those, 15 probes were annotated in GO: 3 were for the major lymphoid-restricted membrane protein (*JAW1*), 2 for the B-cell CLL/lymphoma 6 (*BCL6*), 1 for the B-cell CLL/lymphoma 7A (*BCL7A*), 1 for the Cylin D2. Most of the 15 genes were found to be germinal centre B cell signatures [24], suggesting that *G*_4 _described a complex process leading to a favourable survival outcome for DLBCL patients. However, we recognized that compared with *G*_2 _some new genes (or functions) were identified in *G*_4_, which may define some new pathways or expand our knowledge on the functional topology for DLBCL, or may be simply due to the differences between the two datasets. For the functional annotations for the known genes included in *G*_4_, see Additional File 2.

It is interesting to note that several genes were repeatedly identified as important molecular signatures for DLBCL. For example, multiple probes for *JAW1 *were repeatedly identified in both datasets. In addition, many previous experiments also detected the overexpression of this gene in normal [30] or impaired germinal centre (GC) B-cells [31] and *JAW1 *had thus been established to be one of the most important molecular signatures for the GCB subtype of DLBCL [9,32-34]. The *JAW1 *gene, encoding a lymphoid-restricted membrane protein (hence also called LRMP), was first identified by screening for genes expressed preferentially in B-cell lines [35] and later found in lymphoid tissues and in the pancreas and colon [36]. A recent immunohistological study of B-cell lymphomas [31] documented *JAW1 *expression at the protein level in human tissues by using immunohistochemical and western blotting. And the investigators found that *JAW1*-encoded protein was also highly expressed in germinal centre B-cells. Overall, multiple lines of evidence at different molecular levels support that *JAW1 *is the most important prognostic marker for lymphoma. Interestingly, the *JAW1 *gene has been reported to be fused to the *BLC6 *gene in a case of transformed follicle centre lymphoma [37]. In this study, both genes were included in *G*_4 _for the second dataset.

*BCL6*, *BCL7A*, *KIAA1281*, *Cyclin G2*, *PIK3CG *and *JAW1 *included in the subsets of G2 and/or G4 for the two datasets are previously reported germinal centre-associated signatures [24]. *BCL6 *was the prognostic marker for GCB-like DLBCL and non- GCB-like DLBCL groups and *JAW1 *has different expressions among the three subtypes identified by Wright et al. [32]. Consequently, it is not surprising that both subsets of genes had high discriminating power in recognizing GCB subtype of DLBCL. Based on the definitions of DLBCL subtypes proposed by Alizadeh et al. [9], most of subtype 1 partitioned by *G*_2 _were of germinal centre B-cell-like (GCB-like) DLBCL (17 GCB-like DLBCL cases: 2 activated B-like (AB-like) DLBCL cases), and all of subtype 3 were of AB-like DLBCL. However, the clinicopathological characteristics of subtype 2, consisting of 5 AB-like DLBCL cases and 4 GCB-like cases were less clear. As shown in Figure 1, the subtype 2 defined by *G*_2_, which may correspond to Rosenwald et al's type 3 DLBCL [10], did not express the set of genes of *G*_2 _at a high level.

### Survival analysis

**Figure 3:**
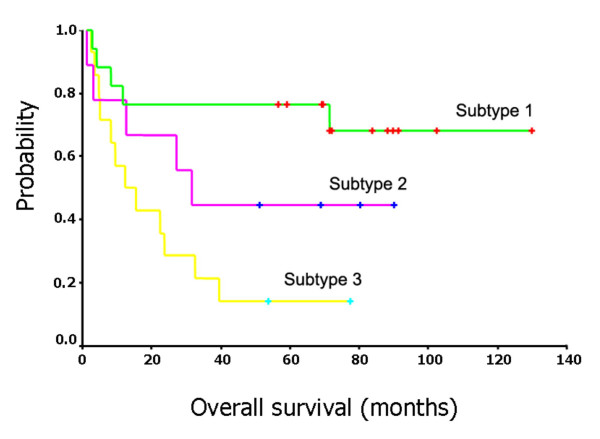
Survival curves for three subtypes of the DLBCL patients in the Alizadeh et al's dataset.

**Figure 4:**
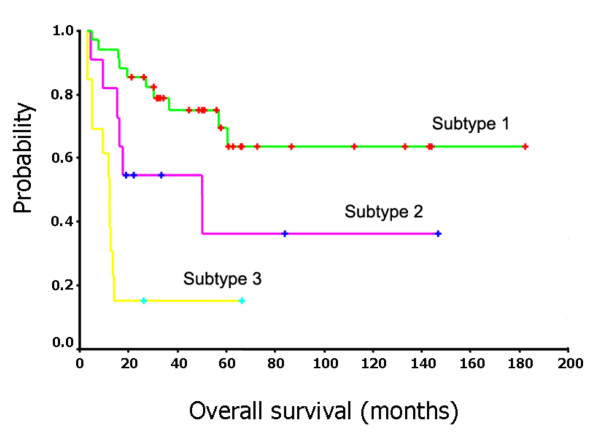
Survival curves for three subtypes of the DLBCL patients in the Shipp et al's dataset.

To verify the clinical significance of the identified hidden DLBCL subtypes, we estimated survival curves by using Kaplan-Meier product-limit method and assessed the differences between the survival curves of the subtypes of DLBCL patients by a log-rank test [38]. For the first dataset, the survival curves associated with the three subtypes revealed by *G*_2 _are shown in Figure 3. The log-rank statistic comparing the survival times of the first subtype and the third subtype (as shown in Figure 1) shows highly significant differences (*p *= 0.0017). The 5 year survival rates for three subtypes were 70.59%, 44.44% and 14.29%, respectively. The survival curves associated with the three subtypes identified by *G*_4 _in the second dataset are similar to the plots for the first dataset except for subtype 3 that has a steeper survival curve (Figure 4). The log-rank statistic comparing the survival times of the first subtype and the third subtype shows highly significant differences (*p *= 0.0009). The 5 year survival rates for three subtypes were 63.13%, 34.92% and 15.38%, respectively.

In order to explore a compact model for clinical use, we further identified the most contributed genes of high prediction power. Multivariate Cox proportional-hazards model was used to analyze the genes in *G*_2 _and *G*_4_, respectively. To reduce the number of variables to be modelled, we applied the stepwise variable selection option (with the same inclusion and exclusion *p *value of 0.05) for the multivariate Cox proportional-hazards regression model [39]. We ended up with four predictors (genes) for *G*_2 _and two predictors for *G*_4_, respectively (Tables 1 and 2). Not surprisingly, both *BCL6 *and *JAW1 *were selected to be the significant prognostic predictors because of their importance involved in the underlying pathogenic mechanisms for lymphoma. Hans et al [40] estimated that it was feasible to assign patients to GCB-like and non-GCB-like groups based on only three markers (*CD10*, *BCL6*, and *MUM1/IRF4*). However, the finding of *JAW1 *being repeatedly selected for both datasets may implicates its discriminating role in lymphoma patients at large. In both datasets, the *JAW1 *gene was defined as an important 'favourable' prognosis predictor (*p *= 0.005 and 0.014; hazard ratios = 0.02 and 0.03, respectively for two datasets). Based on the GO function data, all other three genes (*PRKCBP1*, *TAFII32*, *CCNH*) in *G*_2 _are associated with the functions of 'regulation of transcription', implying their putative roles in cell development and cycling (also see Additional File 1).

**Table 1:** Multivariate Cox proportional-hazards analysis based on the *G*_2 _signature genes relevant to survival time

Variable	Estimated coefficient	Wald χ^2^	*p *value	Hazard ratio (95% CI)
*PRKCBP1*	4.16	9.08	0.003	63.97 (4.28–956.77)
*TAFII32*	4.07	9.16	0.002	58.55 (4.20–817.04)
*CCNH*	4.23	10.40	0.001	68.55 (5.25–895.07)
*JAW1*	-3.86	7.99	0.005	0.02 (0.00–0.16)

**Table 2:** Multivariate Cox proportional-hazards analysis based on the *G*_4 _signature genes relevant to survival time

Variable	Estimated coefficient	Wald χ^2^	*p *value	Hazard ratio (95% CI)
*BCL6*	-3.42	4.58	0.032	0.20 (0.01–0.72)
*JAW1*	-3.03	6.00	0.014	0.03 (0.00–0.48)

## Discussion and conclusion

An initial gene expression profiling study of DLBCL led to the discovery that this single diagnostic category consists of at least two molecularly distinct diseases [9]. One DLBCL subtype, termed GCB-like DLBCL, expressed genes characteristic of normal germinal centre B cells whereas the other subtype, termed AB-like DLBCL, instead expressed genes characteristic of mitogenically activated blood B cells. Patients with GCB-like DLBCL were more often cured by chemotherapy than patients with AB-like DLBCL were. Recently, in an expanded gene expression profiling study of 274 DLBCL patients, the two gene expression subtypes were again identified together with a new subtype, termed type 3, which did not express the genes characteristic of either GCB- or AB-like DLBCL [10]. As before, patients with GCB-like DLBCL had a more favourable clinical course, with a 5-yr survival rate of 60% compared with 5-yr survival rates of 35% and 38% for patients with AB-like and type 3 DLBCL, respectively. Another study used oligonucleotide microarrays to profile gene expression in 58 DLBCL biopsies [24] and attempted to identify the GCB- and AB-like DLBCL subtypes using the genes that were identified in the original profiling study for distinguishing these subtypes [9]. Hierarchical clustering of the DLBCL cases based on expressions of these genes resulted in two groups of patients that did not differ in clinical outcome [24], in apparent contrast with the two other studies [9,10]. Compared with the previous studies, the two-way clustering algorithm applied in this study appears more efficient in finding the most compact gene subsets that have achieved an improved prognostic accuracy over the DLBCL patients' survival profiles. However, it should be cautioned that more detailed clinicopathologic characteristics for subtype 2 DLBCL patients as defined by either gene subset *G*_2 _or *G*_4 _have to be fully characterized before use although the survival profiles for the subtype can be clearly separated from other two subtypes. Based on the gene expression patterns of *G*_2 _(Figure 1), it appears that many genes in subtype 2 patients were inactivated and hence the *G*_2_'s ability in differentiating these samples was significantly lowered.

Computational discoveries of the hidden subtypes for a complex disease have to be verified by some means, e.g., a functional assay using bioinformatics approaches or a clinical validation using epidemiological approaches such as survival analysis. In supervised classification, the choice of the best subset of genes for disease prediction should be relatively easy because the sample labels in training set are given, the high accuracy rate(s) of the classifiers trained on the candidate subsets might be used to filter more specific and critical subsets highly relevant to a disease pathogenesis. In unsupervised clustering analysis, however, identifying the best subset for peeling clinically heterogeneous disease can be a very challenging task as no cross-validation can be done internally. The underlying assumption for a clustering algorithm is that genes with similar expression patterns are more likely to have a similar biological function(s), but a clustering algorithm itself does not provide proof of the best grouping of genes in terms of biological functions [41]. Thus, the biological interpretation of the disease clustering results relies heavily on the expert knowledge which often is somewhat subjective [42]. Therefore, in this study, we designed a functional consensus score for evaluating a candidate gene subset in terms of functional concept consistency, which is similar to the biological homogeneity index (BHI) proposed recently [43]. Based on evaluating the performance of ten well-known clustering algorithms on two gene expression datasets, the authors in [43] found that a good clustering algorithm should have a high BHI. Alternatively, one can use the external annotation database such as Gene Ontology to directly guide the selection of multiple functionally compact and coherent gene subsets (modules) as we did in a recent study [18]. In terms of the better-characterized functionality of subsets *G*_2 _and *G*_4 _and based on the significantly different survival results for the patients defined by the newly defined subtypes, the applied two-way clustering algorithm has been demonstrated to be a feasible and promising toolbox for peeling off molecular heterogeneities of complex human diseases.

In this study, we took the known subtypes suggested by previous studies as the basis to assess the validity of the proposed approach. Although the clustering results provided good fits to these known phenotypic partitions, the implied assumption of the lack of other subtypes or subtle DLBCL groups might not be true. Also, the problem to estimate the correct number of subtypes for peeling off complex diseases is not investigated in this study. Some investigators [44-46] have proposed several methods to obtain the best number of sample partitions by optimizing some validity indices such as the adjusted Rand index (ARI) [44], which would provide additional insights onto improving the two-way clustering algorithm applied in this study.

There is a growing interest in biomedical domains for developing robust predictive model for the survival of cancer patients using gene expression data. However, many methods use all the genes on chips or a large number of genes (e.g. those filtered according to a marginal threshold) to predict a survival. Since the vast majority of the genes in a given dataset are irrelevant to the survivals of the studied patients, the result is that many of the inputs to the predictive model are superfluous and thus reduce the accuracy of the model for prediction. Hence, McLachlan et al. [47] proposed a mixture model-based approach to the clustering of microarray expression data. In this approach, a subset of the genes relevant for the clustering of the tissue samples was first selected by fitting mixtures of t distributions to rank the genes in order of increasing size of the likelihood ratio statistic for the test of one versus two components in the mixture model. Then, if this reduced set of genes is still too large for a normal mixture model to be fitted directly to the tissues, the investigators suggested the use of mixtures of factor analyzers to reduce the dimension of the feature space of genes further. In this study, we applied an integrative approach that combines a SPC-based two-way clustering with a functional consensus metric to identify functionally sounding and the most compact subset of genes underlying the phenotypic partitions of patients. Application of the proposed approach to two DLBCL datasets led to identification of two gene subsets with several features overlapped, and further multivariate Cox proportional-hazards modelling defined *JAW1 *as one of the most significant predictors for the survival of the DLBCL patients in both cohorts. Overall, our results demonstrated that the proposed approach is promising for peeling off the hidden genetic heterogeneity based on modern omics data, and may lead to an improved diagnosis and treatment of cancers.

## Methods

### Super-paramagnetic clustering

SPC is a newly developed clustering method by mimicking the physical attributes of inhomogeneous ferromagnets. A detailed description of the algorithm can be found in [14,48]. Here only a brief introduction to the method is provided. At first, SPC builds a weighted graph for a putative data partition by computing the linkage edge weights of each object and its K nearest neighbours, respectively. Then, it evaluates each data partition using a cost function. Finally, it identifies each cluster through combining each kind of partitions.

#### (1) Weighted graph

For each clustering object data *Z*_*i *_(*i *= 1, ..., *N*), a feature vector that corresponds to a point in a D-dimensional space, we computed the distance: *d*_*ij *_= |*Z*_*i *_- *Z*_*j*_|, (*i*, *j *= 1, ..., *N*). If *Z*_*j *_was one of the *K *closest neighbours of *Z*_*i*_, then we connected the two points *Z*_*i *_and *Z*_*j *_by an edge with a weight:



where α was the average of *d*_*ij*_, and *K *was the number of neighbours for an object. We fixed *K *= 10.

#### (2) Cost function for graph partitions

We randomly assigned an integer label *L*_*i *_= 1, 2, ..., *q *{*L*_1_, *L*_2_, ..., *L*_*N*_} to the *i*-th object to produce a partition {*Z*}. If *L*_*i *_= *L*_*j *_in a partition then *Z*_*i *_and *Z*_*j *_belong to the same cluster *C*. Otherwise, they were in different clusters. For the simulation, we fixed *q *= 20.

The cost function of {*Z*} was:



where



The lowest cost *H*(*Z*) = 0 was obtained when all data points belong to one group; the highest cost was reached if none of its neighbours was from the same group. The smaller distance between two points relates a higher likelihood that they belong to the same group. Hence the value of *H*(*Z*) reflects the resolution at which the partition {*Z*} views the data.

#### (3) Ensemble of partitions

We considered all configurations {*Z*} that had (nearly) the same value of *H*(*Z*) = *E *rather than choosing any particular partition (say by minimizing the cost function). In the resulting statistical ensemble of partitions, each {*Z*} appeared with the statistical weight *P*({*Z*}) ∝ *e*^-*H*(*z*)/*T*^: at *T *= 0 only groupings with *E *= 0 had a non-vanishing weight; at *T *= ∝ all partitions had an equal weight. For a sequence of values of the temperature *T*, we calculated, by Monte Carlo simulation, the average of , the probability of *Z*_*i *_and *Z*_*j *_in the same cluster at the resolution set by *T*.

#### (4) Identifying clusters

The "stable" clusters were discovered under the null hypothesis specified by *R*_*ij *_by following a three-step procedure. First, we built the cluster "core" using threshold *R*_*ij*_. For every pair of neighbours *Z*_*i *_and *Z*_*j*_, if *R*_*ij *_> 0.5, we set a "link" between *Z*_*i *_and *Z*_*j*_. Second, we captured the points lying on the periphery of the clusters by linking each point *Z*_*i *_to its neighbour *Z*_*j *_of the maximal correlation *R*_*ij*_. Third, we identified the data clusters from the linked components of the graphs obtained in the former two steps.

In the SPC procedure, a tuneable parameter *T *("temperature") controlled the resolution of the performed clustering. One started at *T *= 0, all the objects dropped in a single cluster. As *T *increased, this cluster broke into several subclusters that reflected the structure of the data. Clusters kept breaking up as *T *was further increased, until each object formed its own cluster at high enough values of *T*_max_. At last, SPC formed a hierarchical dendrogram.

As opposed to most agglomerative algorithms, SPC had a natural measure of relative stability over a range of temperatures, Δ*T*_*c*_, in which the cluster retained unchanged. The more stable cluster was expected to "survive" over a larger range of Δ*T*_*c*_. For evaluating the stability of a cluster, we set a value for Δ*T*_*c*_, above which a cluster was considered as stable. In order to obtain Δ*T*_*c*_, we randomly permuted elements of the expression matrix under investigation, and applied SPC to the randomized matrix. Δ*T*_*c *_was determined until no clusters satisfied Δ*T*_*c *_> Δ*T *among 500 different random permutations. This gave a bound on the probability that the clusters that we labelled as stable were in fact an artefact of noisy data. For a stable cluster, the larger the range Δ*T*_*c *_was, the more stable it was. Otherwise, if the number of objects involved in a stable cluster was small, SPC considered it as a noisy cluster. Generally, the value is set to be five.

### The CTWC method

The applied CTWC algorithm is a heuristic and iterative method [13]. For a gene expression profile matrix *M*, we denoted the initial sample set as *S*_1_, and the gene set *G*_1_. Clustering gene set *G*_*i *_on the basis of their expression levels over the set of samples *S*_*j *_was referred to the process in an operation denoted by *G*_*i*_(*S*_*j*_). Similarly defined, *S*_*j*_(*G*_*i*_) described the process in clustering *S*_*j *_using all genes of *G*_*i*_. The computational procedures for the CTWC method can be described as follows:

#### (1) Initialization

Compute *S*_1_(*G*_1_) = {*S*_*j*_}, (*j *= 2, 3, ...), and then *G*_1_(*S*_1_) = {*G*_*i*_}, (*i *= 2, 3, ...). Now the cluster depth equals to 0.

#### (2) Identification of stable clusters of genes and samples

Find the most stable *G*_*i *_(*i *= 2, 3, ...) and *S*_*j *_(*j *= 2, 3, ...) per the stability described previously. Compute *S*_*j*_(*G*_*i*_) (including *S*_1_) and *G*_*i*_(*S*_*j*_) (including *G*_1_) for clusters of depth of 1.

#### (3) Iterations

Repeat (2) until the updated clusters were smaller than some fixed threshold or the maximally allowed cluster depth was reached.

### Evaluation of a gene subset by functional concept consistency using GO

GO describes functions of genes and relationships between genes using standard terms. It annotates the functions of a gene from dimensions of molecular function, biological process and cellular component. Generally, genes that take part in a same biological process (such as a metabolism pathway or signal transduction pathway) or that are situated in a proximate subcellular location, often share some function(s) [18].

We obtained many high-correlation sample subsets and gene subsets. In order to identify tumour subtypes both biologically meaningful and clinically relevant, we evaluated a functional concept consistency score for a gene subset to define its biological meanings. The GO-based consistency score was proposed to measure the functional consensus of the entire set of clusters produced by some unsupervised clustering algorithms such as super-parametric two-way clustering used in this study. The aim for developing this metric was to expand current clustering algorithms to produce biological meaningful clusters that are not only able to find the stable partition(s) hidden in the data, but also are useful for elucidating the underlying mechanisms leading to distinct molecular forms in phenotypically defined disease. This index is similar to the biological homogeneity index recently proposed by Datta and Datta [43], measuring how biological homogeneous the clusters are. It may also be considered as a broader and across-GO-node function definition of a cluster of genes, and is particularly useful for evaluating a gene subset that have more than one common function and an included gene is annotated with multiple classes of functions.

The steps for computing a concept consistency score [49] for gene subsets *G*_*i *_(*i *= 1, 2, ..., *N*) were described as follows:

#### (1) Functional annotation

For *g*_*j *_∈ *G*_*i*_, we mapped gene *g*_*j *_to a node(s) of GO via the links between three databases: GeneBank, Unigene, and LocusLink. As a result, we obtained a concept set (*U*_*j *_= {*e*_*j*1_, *e*_*j*2_, ..., *e*_*jm*_}) for gene *g*_*j*_.

#### (2) Distance between concepts

Also, for *g*_*k *_∈ *G*_*i*_, the concept set of *g*_*k*_, *U*_*k *_= {*e*_*k*1_, *e*_*k*2_, ..., *e*_*kn*_} was obtained. We computed the distance between *g*_*j *_and *g*_*k*_:



where

*d*(*e*_*jk*_, *e*_*kl*_) = depth(*e*_*jk*_) + depth (*e*_*kl*_) – 2depth *d*(*e*_*jk*_, *e*_*kl*_)

depth(*e*) denoted the depth of concept *e*, say the distance between *e *and the root of GO, and depth(*e*_*jh*, _*e*_*kl*_) denotes the depth of the nearest common father of the concepts *e*_*jh *_and *e*_*kl*_.

#### (3) Concept consistency

For each *G*_*i*_,



A higher *CC*(*G*_*i*_) corresponds to a higher degree of functional consistency among the genes involved in *G*_*i*_. We knew that the deeper the node of GO was, the more specific the function description was. Ideally, we should consider the hierarchical structure of GO when we computed concept consistency between two nodes. In this study, we added a weight to each concept to improve the similarity estimates between the nodes. The weight of each concept was: , where *d = *depth(*e*). The smaller the weight was, the deeper the concept was. The value of *W*_0 _was assigned to be *W*_0 _= 0.75 in this study.

### Survival analysis

Prior to survival analysis, we obtained the arithmetic mean from the data of multiple probes that correspond to an identical gene. To verify the clinical significance of the identified hidden DLBCL subtypes, we estimated survival curves by Kaplan-Meier product-limit method, and assessed the differences between the survival curves of the subtypes of DLBCL patients by a log-rank test [50]. To construct a model for predicting the overall survival time, a multivariate Cox proportion-hazards model [39] was used to determine the significance (at significant level *p *< 0.05) of the effects of the genes included in the identified gene subset(s) on the patients' survival months. Wald Chi-square test was used to determine the significance of each predictor's hazard toward the survival time.

## Computational algorithms

**Figure 5:**
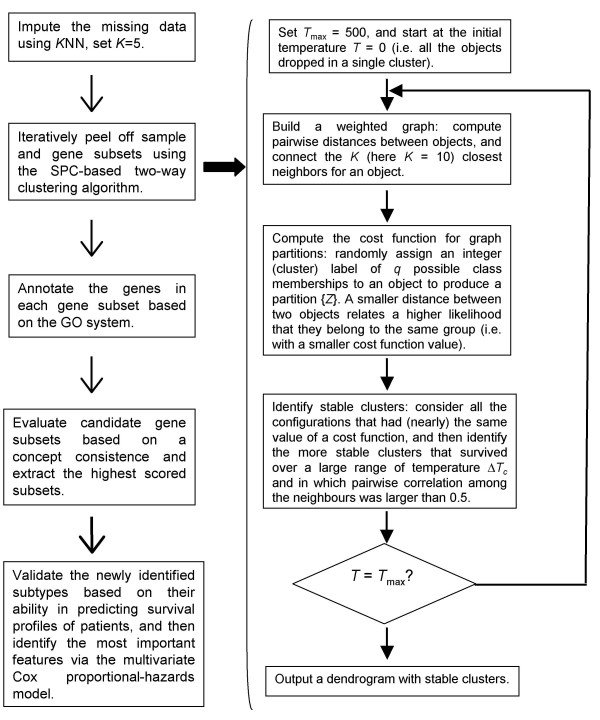
The graphic algorithm flow for the proposed SPC-based two-way clustering.

The algorithm flow for the proposed heterogeneity analysis strategy, organized step-by-step, was graphically depicted in Figure 5. The SPC-based two-way clustering was realized on a public server [26]. The corresponding programming codes for computing a function concept consistency score are available upon a written request to the authors. The hierarchical dendrogram resulted from the coupled two-way clustering was plotted by Treeview [27,28].

## Abbreviations

diffuse large B-cell lymphoma (DLBCL), activated B-like DLBCL (AB-like DLBCL), germinal centre B-like DLBCL (GCB-like DLBCL), coupled two-way clustering (CTWC), super-paramagnetic clustering (SPC), GeneOntology (GO).

## Competing interests

The author(s) declares that there are no competing interests.

## Authors' contributions

This study was undertaken by a collaborative team of several institutes as indicated. WZ, LL, XL and SR conceived of the proposal of the study, conducted the study and drafted the manuscript. The remaining authors participated in writing the computing codes and applied the data mining strategy to the field datasets. All authors participated in reading, approving and revising the manuscript.

## Supplementary Material

Additional file 1 Table S1 – The functional annotations for the known genes included in *G*_2_ Click here for file

Additional file 2 Table S2 – The functional annotations for the known genes included in *G*_4_ Click here for file
