# Supplementary material for: Unravelling the hidden heterogeneities of diffuse large B-cell lymphoma based on coupled two-way clustering
Source: BMC Genomics. 2007 Sep 22;8:332. doi: 10.1186/1471-2164-8-332 (PMC2082044; doi:10.1186/1471-2164-8-332)
Supplement: Additional file 1 — Table S1 – The functional annotations for the known genes included in G2 [file 1471-2164-8-332-S1.doc]

### Additional file 1: Table S1 – The functional annotations for the genes included in *G*_2_

| Gene Symbol | Name | GO:Biological Process | GO:Molecular Function | GO:Cellular Component |
| --- | --- | --- | --- | --- |
| FCRL3 | Fc receptor-like 3 |  | [Receptor activity](http://godatabase.org/cgi-bin/go.cgi?view=details&depth=1&query=GO:0004872) |  |
| PP2CG | Protein phosphatase 2C, gamma isoform | [Cell cycle arrest](http://godatabase.org/cgi-bin/go.cgi?view=details&depth=1&query=GO:0007050); [Protein amino acid dephosphorylation](http://godatabase.org/cgi-bin/go.cgi?view=details&depth=1&query=GO:0006470) | [Hydrolase activity](http://godatabase.org/cgi-bin/go.cgi?view=details&depth=1&query=GO:0016787); [Protein phosphatase type 2C activity](http://godatabase.org/cgi-bin/go.cgi?view=details&depth=1&query=GO:0015071); [Magnesium ion binding](http://godatabase.org/cgi-bin/go.cgi?view=details&depth=1&query=GO:0000287) | [Nucleus](http://godatabase.org/cgi-bin/go.cgi?view=details&depth=1&query=GO:0005634); [Protein serine/threonine phosphatase complex](http://godatabase.org/cgi-bin/go.cgi?view=details&depth=1&query=GO:0008287) |
| PRKCBP1 | protein kinase C binding protein 1 | [Regulation of transcription, DNA-dependent](http://godatabase.org/cgi-bin/go.cgi?view=details&depth=1&query=GO:0006355); [Cell cycle](http://godatabase.org/cgi-bin/go.cgi?view=details&depth=1&query=GO:0007049) | [DNA binding](http://godatabase.org/cgi-bin/go.cgi?view=details&depth=1&query=GO:0003677) |  |
| UBE2H | Ubiquitin-conjugating enzyme E2H (UBC8 homolog, yeast) | [Ubiquitin cycle](http://godatabase.org/cgi-bin/go.cgi?view=details&depth=1&query=GO:0006512) | [Ligase activity](http://godatabase.org/cgi-bin/go.cgi?view=details&depth=1&query=GO:0016874); [Ubiquitin conjugating enzyme activity](http://godatabase.org/cgi-bin/go.cgi?view=details&depth=1&query=GO:0004840); [Ubiquitin-protein ligase activity](http://godatabase.org/cgi-bin/go.cgi?view=details&depth=1&query=GO:0004842) |  |
| TAFII32 | TAF9 RNA polymerase II, TATA box binding protein (TBP)-associated factor, 32kDa | [Regulation of transcription; DNA-dependent](http://godatabase.org/cgi-bin/go.cgi?view=details&depth=1&query=GO:0006355); [Transcription](http://godatabase.org/cgi-bin/go.cgi?view=details&depth=1&query=GO:0006350)  [Transcription initiation](http://godatabase.org/cgi-bin/go.cgi?view=details&depth=1&query=GO:0006352); [Transcription from RNA polymerase II promoter](http://godatabase.org/cgi-bin/go.cgi?view=details&depth=1&query=GO:0006366) | [General RNA polymerase II transcription factor activity](http://godatabase.org/cgi-bin/go.cgi?view=details&depth=1&query=GO:0016251); [DNA binding](http://godatabase.org/cgi-bin/go.cgi?view=details&depth=1&query=GO:0003677); [ATP binding](http://godatabase.org/cgi-bin/go.cgi?view=details&depth=1&query=GO:0005524); [Transcription coactivator activity](http://godatabase.org/cgi-bin/go.cgi?view=details&depth=1&query=GO:0003713); [Protein C-terminus binding](http://godatabase.org/cgi-bin/go.cgi?view=details&depth=1&query=GO:0008022) | [Transcription factor TFIID complex](http://godatabase.org/cgi-bin/go.cgi?view=details&depth=1&query=GO:0005669); [Nucleus](http://godatabase.org/cgi-bin/go.cgi?view=details&depth=1&query=GO:0005634) |
| HERC2 | Hect domain and RLD 2 | [Ubiquitin cycle](http://godatabase.org/cgi-bin/go.cgi?view=details&depth=1&query=GO:0006512); [Intracellular protein transport](http://godatabase.org/cgi-bin/go.cgi?view=details&depth=1&query=GO:0006886) | [Zinc ion binding](http://godatabase.org/cgi-bin/go.cgi?view=details&depth=1&query=GO:0008270); [Guanyl-nucleotide exchange factor activity](http://godatabase.org/cgi-bin/go.cgi?view=details&depth=1&query=GO:0005085); [Ubiquitin-protein ligase activity](http://godatabase.org/cgi-bin/go.cgi?view=details&depth=1&query=GO:0004842) | [Intracellular](http://godatabase.org/cgi-bin/go.cgi?view=details&depth=1&query=GO:0005622) |
| SPRED2 | Sprouty-related, EVH1 domain containing 2 | [Development](http://godatabase.org/cgi-bin/go.cgi?view=details&depth=1&query=GO:0007275); [Regulation of signal transduction](http://godatabase.org/cgi-bin/go.cgi?view=details&depth=1&query=GO:0009966); [Inactivation of MAPK](http://godatabase.org/cgi-bin/go.cgi?view=details&depth=1&query=GO:0000188) | [Protein binding](http://godatabase.org/cgi-bin/go.cgi?view=details&depth=1&query=GO:0005515); [Stem cell factor receptor binding](http://godatabase.org/cgi-bin/go.cgi?view=details&depth=1&query=GO:0005173) | [Membrane](http://godatabase.org/cgi-bin/go.cgi?view=details&depth=1&query=GO:0016020) |
| BCL7A | B-cell CLL/lymphoma 7A |  | [Actin binding](http://godatabase.org/cgi-bin/go.cgi?view=details&depth=1&query=GO:0003779) |  |
| TNFA | tumor necrosis factor-alpha | [Cell-cell signaling](http://godatabase.org/cgi-bin/go.cgi?view=details&depth=1&query=GO:0007267); [Inflammatory response](http://godatabase.org/cgi-bin/go.cgi?view=details&depth=1&query=GO:0006954); [Leukocyte adhesion](http://godatabase.org/cgi-bin/go.cgi?view=details&depth=1&query=GO:0007159)  egulation of transcription; DNA-dependent; [Anti-apoptosis](http://godatabase.org/cgi-bin/go.cgi?view=details&depth=1&query=GO:0006916); [Apoptosis](http://godatabase.org/cgi-bin/go.cgi?view=details&depth=1&query=GO:0006915); [Signal transduction](http://godatabase.org/cgi-bin/go.cgi?view=details&depth=1&query=GO:0007165) | [Tumor necrosis factor receptor binding](http://godatabase.org/cgi-bin/go.cgi?view=details&depth=1&query=GO:0005164) | [Integral to membrane](http://godatabase.org/cgi-bin/go.cgi?view=details&depth=1&query=GO:0016021) |
| PIK3CG | Phosphoinositide-3-kinase, catalytic, gamma polypeptide | [Phosphoinositide 3-kinase complex](http://godatabase.org/cgi-bin/go.cgi?view=details&depth=1&query=GO:0005942) | [Phosphatidylinositol 3-kinase activity](http://godatabase.org/cgi-bin/go.cgi?view=details&depth=1&query=GO:0016303); [Transferase activity](http://godatabase.org/cgi-bin/go.cgi?view=details&depth=1&query=GO:0016740) | [Phosphoinositide 3-kinase complex](http://godatabase.org/cgi-bin/go.cgi?view=details&depth=1&query=GO:0005942) |
| JAW1 | The lymphoid- restricted membrane protein | Hemocyte development; Vesicle fusion; Vesicle targeting | [Endoplasmic reticulum membrane](http://godatabase.org/cgi-bin/go.cgi?view=details&depth=1&query=GO:0005789) | [Integral to plasma membrane](http://godatabase.org/cgi-bin/go.cgi?view=details&depth=1&query=GO:0005887) |
| CCNH | Cyclin H | [Regulation of transcription, DNA-dependent](http://godatabase.org/cgi-bin/go.cgi?view=details&depth=1&query=GO:0006355); [Regulation of cyclin dependent protein kinase activity](http://godatabase.org/cgi-bin/go.cgi?view=details&depth=1&query=GO:0000079); [Transcription](http://godatabase.org/cgi-bin/go.cgi?view=details&depth=1&query=GO:0006350); [DNA repair](http://godatabase.org/cgi-bin/go.cgi?view=details&depth=1&query=GO:0006281) |  | [Nucleus](http://godatabase.org/cgi-bin/go.cgi?view=details&depth=1&query=GO:0005634) |
| LANCL1, | G protein-coupled receptor 69A | [G-protein coupled receptor protein signaling pathway](http://godatabase.org/cgi-bin/go.cgi?view=details&depth=1&query=GO:0007186) | G-protein coupled receptor activity | [Integral to plasma membrane](http://godatabase.org/cgi-bin/go.cgi?view=details&depth=1&query=GO:0005887) |
| CASC1 | Cancer susceptibility candidate 1 |  |  |  |
| EDEM1 | ER degradation enhancer, mannosidase alpha-like 1 |  |  |  |
| XKR6 | XK, Kell blood group complex subunit-related family, member 6 |  |  |  |
