# Supplementary material for: Unravelling the hidden heterogeneities of diffuse large B-cell lymphoma based on coupled two-way clustering
Source: BMC Genomics. 2007 Sep 22;8:332. doi: 10.1186/1471-2164-8-332 (PMC2082044; doi:10.1186/1471-2164-8-332)
Supplement: Additional file 2 — Table S2 – The functional annotations for the known genes included in G4 [file 1471-2164-8-332-S2.doc]

### Additional file 2: Table S2 – The functional annotations for the genes included in *G*_4_

| Gene Symbol | Gene Name | GO:Biological Process | GO:Molecular Function | GO:Cellular Component |
| --- | --- | --- | --- | --- |
| Cyclin G2 | Cyclin G2 | Cell cycle checkpoint; [Regulation of cell cycle](http://godatabase.org/cgi-bin/go.cgi?view=details&depth=1&query=GO:0000074); Cytokinesis [Mitosis](http://godatabase.org/cgi-bin/go.cgi?view=details&depth=1&query=GO:0007067) |  |  |
| BCL6 | B-cell CLL/lymphoma 6 (zinc finger protein 51) | [Positive regulation of cell proliferation](http://godatabase.org/cgi-bin/go.cgi?view=details&depth=1&query=GO:0008284); [Regulation of transcription, DNA-dependent](http://godatabase.org/cgi-bin/go.cgi?view=details&depth=1&query=GO:0006355); [Transcription](http://godatabase.org/cgi-bin/go.cgi?view=details&depth=1&query=GO:0006350); [Inflammatory response](http://godatabase.org/cgi-bin/go.cgi?view=details&depth=1&query=GO:0006954); [Negative regulation of transcription from RNA polymerase II promoter](http://godatabase.org/cgi-bin/go.cgi?view=details&depth=1&query=GO:0000122) | [Protein binding](http://godatabase.org/cgi-bin/go.cgi?view=details&depth=1&query=GO:0005515); [Transcription factor activity](http://godatabase.org/cgi-bin/go.cgi?view=details&depth=1&query=GO:0003700); [Zinc ion binding](http://godatabase.org/cgi-bin/go.cgi?view=details&depth=1&query=GO:0008270) | [Nucleus](http://godatabase.org/cgi-bin/go.cgi?view=details&depth=1&query=GO:0005634); [Mediator complex](http://godatabase.org/cgi-bin/go.cgi?view=details&depth=1&query=GO:0000119) |
| Jaw1 | The lymphoid- restricted membrane protein | [Hemocyte development](http://godatabase.org/cgi-bin/go.cgi?view=details&depth=1&query=GO:0007516); [Vesicle fusion](http://godatabase.org/cgi-bin/go.cgi?view=details&depth=1&query=GO:0006906); [Vesicle targeting](http://godatabase.org/cgi-bin/go.cgi?view=details&depth=1&query=GO:0006903) | [Endoplasmic reticulum membrane](http://godatabase.org/cgi-bin/go.cgi?view=details&depth=1&query=GO:0005789) | [Integral to plasma membrane](http://godatabase.org/cgi-bin/go.cgi?view=details&depth=1&query=GO:0005887) |
| Wnt10B | Wingless-type MMTV integration site family, member | [Signal transduction](http://godatabase.org/cgi-bin/go.cgi?view=details&depth=1&query=GO:0007165); [Development](http://godatabase.org/cgi-bin/go.cgi?view=details&depth=1&query=GO:0007275); [Frizzled-2 signaling pathway](http://godatabase.org/cgi-bin/go.cgi?view=details&depth=1&query=GO:0007223) | [Signal transducer activity](http://godatabase.org/cgi-bin/go.cgi?view=details&depth=1&query=GO:0004871) | [Extracellular region](http://godatabase.org/cgi-bin/go.cgi?view=details&depth=1&query=GO:0005576) |
| CRYAA | Crystallin, alpha A | [Protein folding](http://godatabase.org/cgi-bin/go.cgi?view=details&depth=1&query=GO:0006457); [Sensory perception](http://godatabase.org/cgi-bin/go.cgi?view=details&depth=1&query=GO:0007600); [Visual perception](http://godatabase.org/cgi-bin/go.cgi?view=details&depth=1&query=GO:0007601) | [Unfolded protein binding](http://godatabase.org/cgi-bin/go.cgi?view=details&depth=1&query=GO:0051082); [Structural constituent of eye lens](http://godatabase.org/cgi-bin/go.cgi?view=details&depth=1&query=GO:0005212) |  |
| FDXR | Ferredoxin reductase | [Lipid metabolism](http://godatabase.org/cgi-bin/go.cgi?view=details&depth=1&query=GO:0006629); [Steroid biosynthesis](http://godatabase.org/cgi-bin/go.cgi?view=details&depth=1&query=GO:0006694); [Transport](http://godatabase.org/cgi-bin/go.cgi?view=details&depth=1&query=GO:0006810); [Cholesterol metabolism](http://godatabase.org/cgi-bin/go.cgi?view=details&depth=1&query=GO:0008203); [Electron transport](http://godatabase.org/cgi-bin/go.cgi?view=details&depth=1&query=GO:0006118) | [Ferredoxin-NADP+ reductase activity](http://godatabase.org/cgi-bin/go.cgi?view=details&depth=1&query=GO:0004324); [Oxidoreductase activity](http://godatabase.org/cgi-bin/go.cgi?view=details&depth=1&query=GO:0016491) | [Mitochondrion](http://godatabase.org/cgi-bin/go.cgi?view=details&depth=1&query=GO:0005739) |
| Bcl7A | B-cell CLL/lymphoma 7A |  | [Actin binding](http://godatabase.org/cgi-bin/go.cgi?view=details&depth=1&query=GO:0003779) |  |
| CYP-60 | Peptidylprolyl isomerase (cyclophilin)-like 2 | [Protein folding](http://godatabase.org/cgi-bin/go.cgi?view=details&depth=1&query=GO:0006457) | [Peptidyl-prolyl cis-trans isomerase activity](http://godatabase.org/cgi-bin/go.cgi?view=details&depth=1&query=GO:0003755); [Isomerase activity](http://godatabase.org/cgi-bin/go.cgi?view=details&depth=1&query=GO:0016853) | [Nucleus](http://godatabase.org/cgi-bin/go.cgi?view=details&depth=1&query=GO:0005634) |
